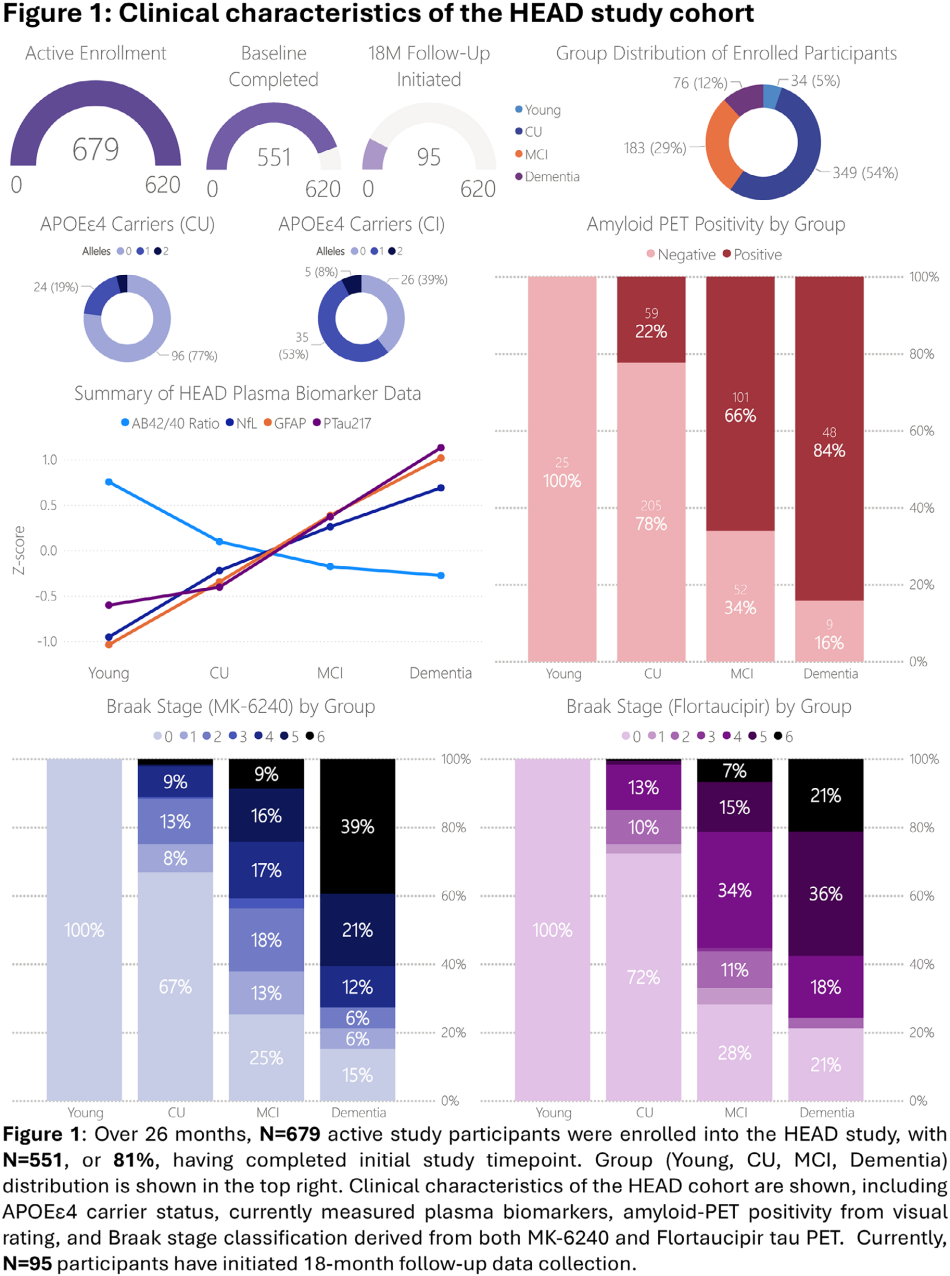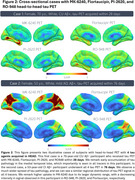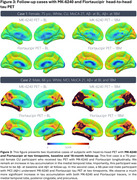# Longitudinal multicenter head‐to‐head harmonization of tau‐PET tracers

**DOI:** 10.1002/alz70862_110115

**Published:** 2025-12-23

**Authors:** Firoza Z Lussier, Guilherme Povala, Guilherme Bauer‐Negrini, Pamela C.L. Ferreira, Bruna Bellaver, Livia Amaral, Juli Cehula, Joseph C. Masdeu, Thomas K Karikari, Dana L Tudorascu, David N. soleimani‐Meigooni, Hwamee Oh, Juan Fortea, Val J Lowe, Belen Pascual, Brian A. Gordon, Pedro Rosa‐Neto, Suzanne L. Baker, Tharick A Pascoal

**Affiliations:** ^1^ University of Pittsburgh, Pittsburgh, PA USA; ^2^ Houston Methodist Research Institute, Houston, TX USA; ^3^ University of California, San Francisco, San Francisco, CA USA; ^4^ Brown University, Providence, RI USA; ^5^ Sant Pau Memory Unit, Hospital de la Santa Creu i Sant Pau, Biomedical Research Institute Sant Pau, Barcelona Spain; ^6^ Mayo Clinic, Rochester, MN USA; ^7^ Washington University School of Medicine, St. Louis, MO USA; ^8^ McGill University, Montreal, QC Canada; ^9^ Lawrence Berkeley National Laboratory, Berkeley, CA USA

## Abstract

**Background:**

Standardizing tau pathology quantification in vivo is challenged by inherent characteristics of tau‐PET tracers. The HEAD study aims to generate a leading, longitudinal head‐to‐head dataset of MK‐6240, Flortaucipir, RO948, and PI‐2620 tau‐PET to harmonize tracers' outcomes and develop tools to generalize findings across studies and trials. Here, we provide an update on the progression of the HEAD study.

**Method:**

HEAD is a multicentric study comprising nine performance sites. Recruitment aimed for 620 individuals between 18‐28 or 50‐90 years, classified as Young/CU/MCI/Dementia. The HEAD protocol involves clinical assessment utilizing the NACC Uniform Data Set, blood collection for banking of plasma/serum/buffy coat/whole blood, and MRI acquisition based on ADNI4. All participants undergo amyloid‐PET with either PiB/NAV4694/Florbetaben/Flutemetamol. All undergo head‐to‐head tau‐PET with at least two tracers, including MK‐6240 (90‐110), Flortaucipir (80‐100), PI‐2620 (45‐75), and RO948 (70‐90). PET data is reconstructed and processed uniformly similarly to ADNI4. The Laboratory of Neuroimaging (LONI) provides centralized databasing, and the National Centralized Repository for ADRD (NCRAD) provides the blood biorepository for all samples. All study procedures are repeated at 18 months.

**Result:**

Over 26 months, *N* = 679 participants were enrolled into HEAD, exceeding our proposed enrollment by 9.5%. Mean age of older adults is 72.1 years, female distribution is 54%, and 24% of individuals are from underrepresented groups (race/ethnicity/rurality). Progression in data collection has led to *N* = 551 (81%) of enrolled participants having a completed initial timepoint, and 1,489 total acquired head‐to‐head tau‐PET scans (mean acquisition window=34.9 days). Clinical characteristics including group distribution, APOEε4 carriership, plasma biomarker distribution (Aβ42/40 ratio/NfL/GFAP/PTau217), consensus visual rating of amyloid‐PET, and Braak stage classification are summarized in Figure 1. Two representative cases (CU/AD) of head‐to‐head tau‐PET with four tau tracers are shown in Figure 2. Longitudinal data collection has been initiated in *N* = 95 participants. Figure 3. demonstrates two cases (CU/MCI) of 18‐month longitudinal head‐to‐head tau‐PET with MK‐6240 and Flortaucipir.

**Conclusion:**

The HEAD study cohort represents a continued effort in the optimization of AD imaging biomarkers. Cross‐sectional and longitudinal data collection in HEAD are ongoing, in addition to comprehensive plasma biomarker measurements. Generation of findings from HEAD cohort data will provide novel and crucial guidance on the use of tau‐PET tracers.